# The Short-Term Prediction of Length of Day Using 1D Convolutional Neural Networks (1D CNN)

**DOI:** 10.3390/s22239517

**Published:** 2022-12-06

**Authors:** Sonia Guessoum, Santiago Belda, Jose M. Ferrandiz, Sadegh Modiri, Shrishail Raut, Sujata Dhar, Robert Heinkelmann, Harald Schuh

**Affiliations:** 1 UAVAC, Department of Applied Mathematics, Universidad de Alicante, Carretera San Vicente del Raspeig s/n, 03690 San Vicente del Raspeig, Alicante, Spain; 2 Department Geodesy, Federal Agency for Cartography and Geodesy (BKG), 60322 Frankfurt am Main, Germany; 3 GFZ German Research Centre for Geosciences, 14473 Potsdam, Germany; 4 Institute for Geodesy and Geoinformation Science, Technische Universität Berlin, 10623 Berlin, Germany; 5 Indian Institute of Technology Kanpur, Kanpur 208 016, Uttar Pradesh, India

**Keywords:** one-dimensional convolutional neural networks, 1D CNN, length of day, atmospheric angular momentum, AAM function, prediction

## Abstract

Accurate Earth orientation parameter (EOP) predictions are needed for many applications, e.g., for the tracking and navigation of interplanetary spacecraft missions. One of the most difficult parameters to forecast is the length of day (LOD), which represents the variation in the Earth’s rotation rate since it is primarily affected by the torques associated with changes in atmospheric circulation. In this study, a new-generation time-series prediction algorithm is developed. The one-dimensional convolutional neural network (1D CNN), which is one of the deep learning methods, is introduced to model and predict the LOD using the IERS EOP 14 C04 and axial Z component of the atmospheric angular momentum (AAM), which was taken from the German Research Centre for Geosciences (GFZ) since it is strongly correlated with the LOD changes. The prediction procedure operates as follows: first, we detrend the LOD and Z-component series using the LS method, then, we obtain the residual series of each one to be used in the 1D CNN prediction algorithm. Finally, we analyze the results before and after introducing the AAM function. The results prove the potential of the proposed method as an optimal algorithm to successfully reconstruct and predict the LOD for up to 7 days.

## 1. Introduction

The Earth’s orientation in space can be expressed through three independent angles (e.g., the Euler angles). However, five Earth orientation parameters (EOP) are generally used, the polar motion (PM), which are the x and y parameters; the diurnal rotation (e.g., ERA = Earth rotation angle or UT1−UTC); and the celestial pole offsets, dX and dY, which describe the adjustments to the conventional IAU precession-nutation models. These parameters provide the rotation of the International Terrestrial Reference System (ITRS) to the Geocentric Celestial Reference System (GCRS) as a function of time.

The EOP can be accurately observed with modern high-precision space geodetic techniques (i.e., very-long-baseline interferometry (VLBI), satellite laser ranging (SLR), global navigation satellite systems (GNSS)) [1,2,3]. Highly accurate celestial pole offsets (CPO) and UT1 are exclusively obtained using VLBI, whereas satellite-based techniques can provide polar motion with high accuracy (up to 50–100 μas) and temporal resolution [4]. The complexity of the data processing of advanced geodetic techniques makes it difficult to determine the EOP in real time. Additionally, short-term EOP predictions are needed for many applications, including the precise tracking and navigation of interplanetary spacecraft missions, laser ranging to satellites and the Moon, and climate forecasting. Consequently, accurate and novel EOP prediction methods combining existing geophysical phenomena information are of critical importance and required by the scientific community [5].

The LOD is the difference between the duration of the day measured using space geodesy and the nominal day of 86,400 s duration and is defined as $LOD=-\frac{d}{dt}\left(UT_{1}-UTC\right)$ [6]. Advanced geodetic techniques can estimate the LOD with high accuracy of up to 5–10 μs [4]. As with the previous parameters, short-term LOD predictions have to be provided for many real-time applications such as interplanetary navigation and precise orbit determination because of its coupling with the orbit node.

The LOD is the most challenging parameter to forecast since the greatest difficulties come from the occurrence of extreme events such as El Nino, which have demonstrated themselves in LOD signals [7,8,9]. The changes in the LOD could be due to tidal or non-tidal origins. Since tidal variations in the LOD can be precisely modeled, they can be removed from LOD data. Subsequently, the tidal term can be considered in the methodology of calculating the LOD prediction. In addition, non-tidal changes in the LOD of periods of five years or less are predominantly initiated by the exchange of angular momentum between the solid Earth and the global atmosphere [4].

Various algorithms have been created to improve the accuracy of LOD predictions such as the auto-covariance (AC) [10,11], wavelet decomposition [12], neural network (NN), [4,13,14,15], combination of least squares and autoregression (LS+AR), and autoregression moving average (ARMA) algorithms [11,16,17], among others. In addition to these examples, other approaches use a direct combination of LOD data and the axial component of the effective angular momentum (EAMz) [6,18,19,20,21,22,23]. Ref. [6] showed that the use of atmospheric angular momentum (AAM) wind terms in the Kalman filter technique could improve short-term LOD predictions. A comparison of LOD predictions computed using a combination of Copula-based analysis and singular spectrum analysis (SSA) illustrated a prediction with few errors over 10 days [5]. In [19], the authors used $UT_{1}$-like observations determined by the AAM in the $UT_{1}-UTC$ combination solution to predict $UT_{1}$, which showed a prediction with few errors compared to the previous forecast strategy [24]. In addition, the prediction method proposed by [23] used 6-day predicted EAM values for the PM and $UT_{1}-UTC$ prediction using LS extrapolation and the AR model.

The most significant difficulties in time-series predictions are due to the temporal dependence between the observations. Therefore, specialized handling of the data is required when fitting and evaluating models. In addition, LOD data contain complex nonlinear factors that vary considerably. In this work, we introduce one of the deep learning methods to improve short-term LOD predictions. One-dimensional convolutional neural networks, hereafter called 1D CNNs, provide powerful capabilities for accurate LOD forecasting. This method is robust to different types of noise present in the input datasets and the mapping function, providing accurate predictions and learning linear and nonlinear relationships despite the presence of missing values [25]. In this study, we use a combination of LS and 1D CNN as a deterministic-stochastic tool for LOD prediction, where the deterministic part is estimated using LS and 1D CNN is used for modeling the stochastic part. We introduce the AAM to the 1D CNN model since it strongly correlates to the LOD. First, we detrend the LOD and Z-component series using the LS method. Then, we obtain the residual series of each one to be used in the 1D CNN prediction algorithm. Multiple sets of ultra-short-term LOD predictions (up to 7 days) are based on the IERS EOP 14 C04 time series. The results show that the proposed approach can efficiently predict the LOD. The remainder of the paper is structured as follows. Section 2 describes the theoretical framework of the algorithm. Section 3 provides a description of the dataset and the followed prediction methodology. The results of the combined method are presented/evaluated in Section 4 in terms of accuracy. The conclusions and future work ideas are finally presented in Section 5.

## 2. Methods

### 2.1. One-Dimensional Convolutional Neural Networks (1D CNN)

A convolutional neural network (CNN) is a type of neural network that is designed to handle image data efficiently. It is a deep feed-forward network that extends a “classic” artificial neural network by adding more layers (deep learning) including the introduction of convolution blocks. The term “convolutional” comes from the implementation of convolution blocks in the network. The first appearance of this type of neural network dates back to 1980, when Kunihiko Fukushima [26] introduced the concept of convolution and downsampling layers, and to 1990, when LeCun et al. published a paper presenting the principles of the CNN modern framework [27,28]. The ability of CNNs to learn and automatically extract features from raw input data can be applied to time-series forecasting problems. A sequence of observations can be treated like one-dimensional image data that a CNN model can read and distill into the most salient elements. CNNs have the benefits of multilayer perceptrons for time-series forecasting, namely support for multivariate inputs and outputs and the ability to learn arbitrarily complex functional relationships, but they do not require that the model learns directly from lag observations. Instead, the model can learn a representation from a large input sequence that is most relevant to the prediction problem.

#### 2.1.1. The Use of 1D CNNs

The input data for CNNs can have a 1D, 2D, or 3D format. For many years, 2D CNNs have been used for image data. Their use has dramatically improved image processing (including image classification, image semantic segmentation, and object detection) [29]. Nevertheless, the number of published articles on 1D CNNs is pretty narrow when compared with 2D CNNs. The 1D signals in 1D CNNs have made various successful contributions, such as electrocardiograms (ECG), electroencephalograms (EEG), electromyograms (EMG) [30,31], and other healthcare applications [32]. A 1D CNN is also used for streamflow prediction [33].

#### 2.1.2. The Architecture of 1D CNN Model

**Figure 1:**
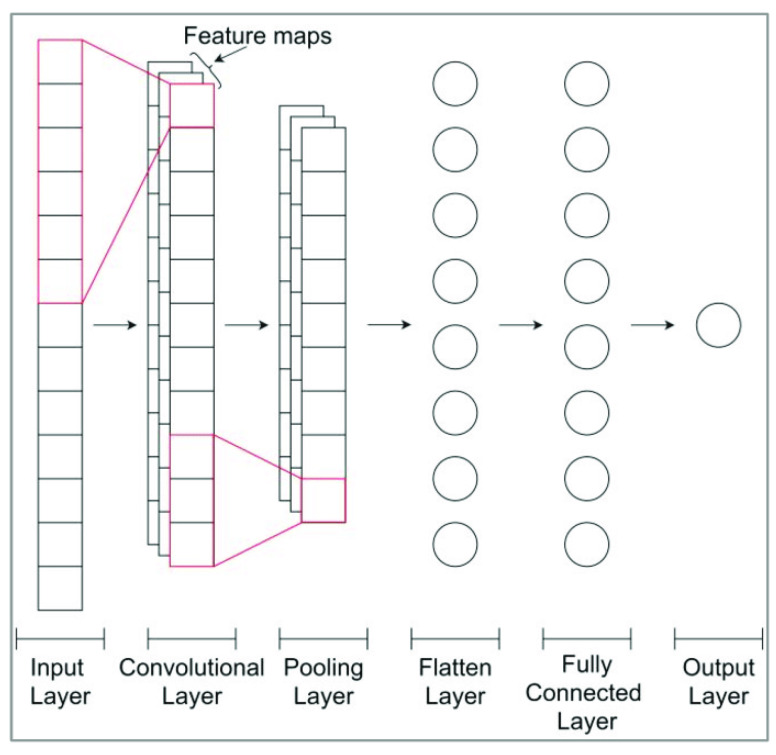
One-dimensional convolutional neural network (1D CNN) architecture.

The typical structure of a 1D CNN is composed of three important layers: 1D convolutional layers, pooling layers, and fully connected layers (see Figure 1) [34]. In addition to these three layers, there are two more important parameters, which are the dropout layer and activation function.

- One-dimensional convolutional layer [35]: This is the layer that can be used to detect features in a vector. The raw one-dimensional input (vector) $x\left[n\right]$, where n=0,1,…,N−1 is given as an input to the first layer of the CNN architecture. The layer utilizes the following parameters:
  1. Filters or kernels: The feature maps are the outputs of one filter applied to the previous layer. The filters/kernels produce the feature maps by performing convolutions with the input data. The number and size of the kernels are crucial for adequately capturing the relevant features from the input data. Let $\kappa \left[n\right]$ denote the convolution kernel with size ϑ, then, the convolution output $\zeta \left[n\right]$ can be calculated as
  (1) $$\begin{array}{cc} \zeta \left[n\right] & =x\left[n\right]*\kappa \left[n\right]=\sum_{m=0}^{\vartheta -1}\kappa \left[m\right].x\left[n-m\right],n=0,1,\ldots ,N-1 \end{array}$$
  where ’*’ denotes the convolution operation. In general, the convolved features at the output of the $l^{th}$ layer can be written as
  (2) $$\begin{array}{c} \zeta_{i}^{l}=\sigma \left(b_{i}^{l}+\sum_{j}\zeta_{j}^{l-1}.\kappa_{ij}^{l}\right) \end{array}$$
  where $\zeta_{i}^{l}$ represents the $i^{th}$ feature in the $l^{th}$ layer, $\zeta_{j}^{l-1}$ denotes the $j^{th}$ features in the ${\left(l-1\right)}^{th}$ layer, $\kappa_{ij}^{l}$ represents the kernel linked from the $i^{th}$ to the $j^{th}$ features, $b_{i}^{l}$ denotes the bias for these features, and σ represents the activation function.
  2. Activation function (R) [35]: One of the most important parameters of the CNN model is the activation function, which is used to learn and approximate any kind of continuous and complex relationship between the variables of the network. There are several activation functions such as the RELU, softmax, and sigmoid functions. In this work, we use the exponential linear unit or its widely known name the ELU, which is an activation function based on the RELU that has an extra alpha constant (α) that defines function smoothness when the inputs are negative. It is a function that tends to converge cost to zero faster and produces more accurate results. Its formula is with α>0:
  (3) $$R\left(\zeta \right)=\left\{\begin{array}{cc} \zeta & \;\mathrm{if}\;\zeta >0\\ \alpha .\left(\exp(\zeta )-1\right) & \;\mathrm{if}\;\zeta <=0 \end{array}\right.$$
- Strid [36]: The strid value defines how the kernel moves in the input data. The most common value is 1, meaning that the kernel moves over one column of the input data at each iteration.
- Pooling Layer [35]: This type of layer is often placed after the convolutional layer. The aim of this layer is to decrease the size of the convolved features map to reduce computational costs. There are several types of pooling operations (max pooling, average pooling, sum pooling) [36]. In this work, we used 1D max pooling, which consists of running the input with a defined spatial neighborhood or specified pool size and strid, taking the maximum value from the considered region. Its operation can be represented by
  (4) $$\begin{array}{c} \zeta_{h}^{l}=\max_{\forall p\in rh}\zeta_{p}^{l-1} \end{array}$$
  where *rh* denotes the pooling region with index *h*.
- Flatten layer and dropout layer [35]: The flatten layer transforms the input data into a one-dimensional vector to be fed to the fully connected/dense layer. A dropout parameter is added after the flatten layer; however, when all the features are connected to the flatten layer, it can cause overfitting in the training dataset. To overcome this problem, a dropout layer is utilized, wherein a few neurons are dropped from the neural network during the training process, resulting in the reduced size of the model.
- Dense fully connected layer [35]: The flattened output is given as an input to the next layer, i.e., the dense fully connected layer, which produces the output. The activation function is one of its parameters. In this work, we use the ELU function, which is described in (4).

### 2.2. Error Analysis

The mean absolute error (MAE) is used to evaluate the performance of the prediction accuracy. We use the MAE instead of the root mean squared error (RMSE) because the MAE is the parameter used by most authors when addressing EOP predictions [37], at least since the first EOP PCC, and it is also the parameter of choice in the EOP PCC2, which can be accessed at http://eoppcc.cbk.waw.pl/ (accessed on 2 September 2022). In addition, several papers have proved that using the MAE is a more natural measure of the average error [38,39]. The MAE is calculated for the $k^{th}$ day in the future as follows:

(5) $$\begin{array}{c} MAE=\frac{1}{k}\sum_{i=1}^{k}\left(|P_{i}-O_{i}|\right) \end{array}$$

where $P_{i}$ is the predicted value of the ith prediction day, $O_{i}$ is the corresponding observed value, and *k* is the total prediction number.

## 3. Calculation and Analysis

### 3.1. Dataset Description

#### 3.1.1. Length of Day (LOD)

In this process, daily time-series data of the LOD were taken from the International Earth Rotation and Reference Systems Service (IERS) combined with the EOP solution 14 C04. This product can be accessed at https://hpiers.obspm.fr/iers/series/opa/eopc04R_IAU2000_dialy (accessed on 2 September 2022). Since older data have much higher uncertainty than more recent data, we excluded them. Hence, we used data from 1999 to 2020.

#### 3.1.2. Atmospheric Angular Momentum (AAM) Function

The mass and motion terms from the AAM models explain the non-tidal geophysical excitation of the Earth’s rotation due to atmospheric mass redistribution. The AAM data consist of three main components X, Y, and Z. The X and Y components are associated with the excitation of polar motion, whereas the Z component plays an important role in interannual LOD variations [23,40,41,42]. Consequently, the latter component was introduced into the proposed prediction since it is strongly correlated with the LOD change using the available model-based EAM for the atmosphere provided by the German Research Centre for Geosciences (GFZ).

### 3.2. Introducing AAM to LOD Prediction Using 1D CNN

**Figure 2:**
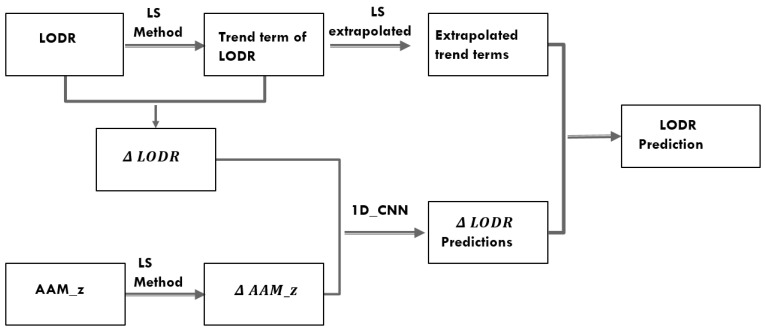
Flowchart of introducing the AAM to LODR predictions using 1D CNN.

In this paper, we define a workflow algorithm to predict the time-varying behaviors of the LOD change (Figure 2). Changes in the LOD can be of tidal or non-tidal origin since tidal variations, such as the influence of the solid Earth tides of periods of 5 days up to 18.6 years and diurnal and semi-diurnal variations due to ocean tides, can be accurately modeled; therefore, they can be removed from the LOD [43]. These tidal variations were first removed from the observed LOD measurements using the zonal and ocean tide models recommended in the IERS conventions 2010 [43]. The time series obtained after removing these tides are denoted as LODR.

#### 3.2.1. Detrending of LODR

**Figure 3:**
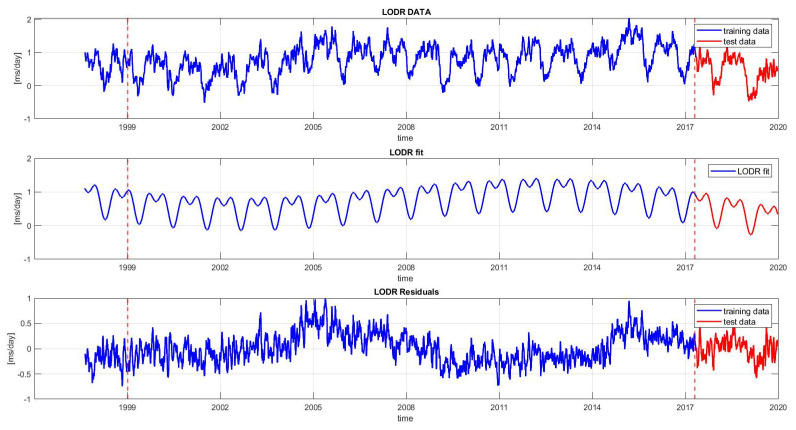
Time series of the LODR between 1999 and 2020. From top to bottom, the data series shown are the LODR, trend terms, and LODR residual series. The time series is divided into two sets: the training set (1999–2017) and testing set (2017–2020).

**Figure 4:**
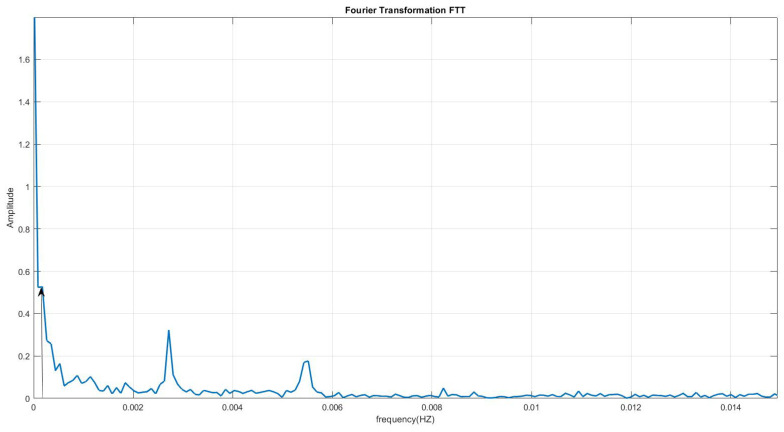
Spectral analysis of the LODR using fast Fourier transform (FFT).

The LODR still includes a linear part and some seasonal variations such as annual and semi-annual oscillations (Figure 3), which are evidenced by the spectral analysis of the LODR illustrated in Figure 4. The parameters of the linear term and seasonal variations are estimated using the LS method in the following equation:

(6) $$\begin{array}{c} f_{LODR}\left(t\right)=\alpha_{0}+\alpha_{1}t+A_{1}\sin\left(w_{1}t+\phi_{1}\right)+A_{2}\sin\left(w_{2}t+\phi_{2}\right)+A_{3}\sin\left(w_{3}t+\phi_{3}\right) \end{array}$$

where $\alpha_{0}$ is the bias, $\alpha_{1}$ is the drift of the linear term, $w_{1}=\frac{2\pi }{365.24}$, $w_{2}=\frac{2\pi }{182.62}$, and $w_{3}=2pi\cdot 1.19\cdot 10^{-04}$.

The LS model is used for two purposes: to obtain stochastic residuals (the difference between the LODR data and LS model) and predict the deterministic components of the signal [4]. The linear and harmonic terms are removed from the LODR in order to avoid the error coming from the extrapolation problem (see Figure 3). Thus, the final prediction of the LODR is the sum of the prediction of the LODR residual series and the extrapolated trend terms. In Figure 3, it can be seen that the LODR residual series still includes some periodic terms, which are modeled later using the 1D CNN.

#### 3.2.2. Detrending of AAM Z-Component Series

**Figure 5:**
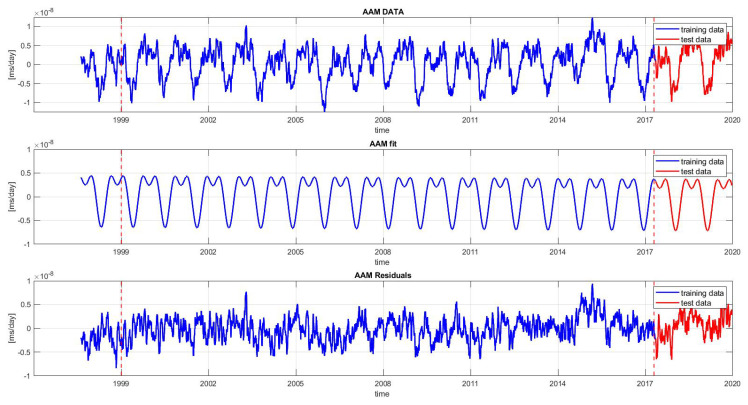
Time series of AAM between 1999 and 2020. From top to bottom, the data series shown are the AAM, trend terms, and AAM Z residual series. The time series is divided into two sets: the training set (1999–2017) and testing set (2017–2020).

Similar to the LODR (previous section), the linear and periodic terms of the AAM Z component were also approximated using the LS method in the same temporal domain (Figure 5). The LS model can be written as

(7) $$\begin{array}{c} f_{AAM}\left(t\right)=A+\beta t+d_{1}\sin\left(w_{1}t+\theta_{1}\right)+d_{2}\sin\left(w_{2}t+\theta_{2}\right) \end{array}$$

where (*A*) is the bias, (β) is the drift of the linear term, and $w_{1}=\frac{2\pi }{365.24}$, $w_{2}=\frac{2\pi }{182.62}$.

#### 3.2.3. LODR Prediction Using 1D CNN

Convolutional neural network models or CNNs can be used for multistep time-series forecasting. CNNs can be used in either a recursive or direct forecast strategy, where the model makes one-step predictions and outputs are fed as inputs for subsequent predictions, and where one model is developed for each time step to be predicted. Alternately, CNNs can be used to predict the entire output sequence as a one-step prediction of the entire vector. This is a general benefit of feed-forward neural networks. An important secondary benefit of using CNNs is that they can support multiple 1D inputs in order to make a prediction. This is useful if the multistep output sequence is a function of more than one input sequence. This can be achieved using two different model configurations [25]:

- Multiple Input channels. This is where each input sequence is read as a separate channel like the different channels of an image (red, green, blue)
- Multiple Input Heads. This is where each input sequence is read by a different CNN submodel and the internal representations are combined before being interpreted and used to make a prediction [25].

In this contribution, we developed a convolutional neural network for multistep time-series forecasting using only the univariate sequence of daily LODR residuals. The number of prior days used as input defines the one-dimensional (1D) subsequence of data. The CNN reads and learns to extract features. The closer the observational data to the day to be forecasted, the greater the impact on the prediction. In addition, it turns out that for near-term predictions, the values from the past few days are the most essential [4]. Consequently, a more sophisticated strategy is to utilize previous values as inputs of the 1D CNN: seven days, two weeks, and one month (28 days).

As shown in Figure 3, the training interval used to feed the 1D CNN was from 1999 to 2017 and the data from 2017 to 2020 were used as the testing set. In the first step, we used the prior seven days.

**Figure 6:**
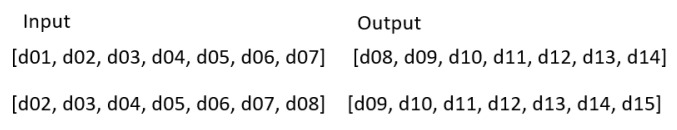
Example of overlapping weekly data.

A 1D CNN model expects data to have the shape of [sample, timestep, features]; one sample will comprise seven timesteps with one feature for the seven days of total daily LODR residuals. Thus, the training dataset was x = [939, 7, 1], meaning that we had 939 weeks of data. The data in this format permitted the model to use the prior standard week to predict the next standard week. A way to create more training data is to change the problem during training to predict the next seven days given the previous seven days, regardless of the standard week. Thus, we iterated over the timesteps and divided the data into overlapping windows; each iteration moved along one timestep and predicted the subsequent seven days (see Figure 6). Therefore, we transformed 939 weeks into 6559. The transformed dataset had the shape x = [6559, 7, 1] For testing our prediction method, we predicted 3 years, which meant 52 weeks per year, which was in total, 156 points for each day of the week.

This multistep time-series forecasting problem is an autoregression, where the next seven days are some function of the observations of the previous seven days. We used a model with one convolutional layer with 64 filters and a kernel size of 4 so the input sequence of seven days was read with a convolutional operation of four time steps at a time and this operation was performed 64 times. The output layer predicted the next seven days in the sequence. We used the mean squared error loss function and the efficient Adam implementation of the stochastic gradient descent and fit the model for 1000 epochs, with a batch size of 32. We made the prediction using only the true observation. The model was required to make a one-week prediction, then, the actual data for that week were computed, which were used as the basis for the prediction of the subsequent week.

#### 3.2.4. Introducing AAM Z-Component Series to LODR Using 1D CNN

In this approach, we combined the AAM Z component with the LODR residual series. In order to achieve this, we developed a multiheaded CNN model to use each of the two time-series variables to predict the next standard week of the LODR. We provided each one-dimensional time series to the model as a separate sub-CNN model (head for each input variable) and the output of each of these sub-models was merged into one long vector, which was interpreted before the prediction was made for the output sequence, representing the prediction of the LODR residuals. We obtained the final prediction of the LODR by adding the LODR trend series extrapolated by Equation (5). In order to understand the prediction performance using different prior days, several prediction models were estimated using the prior 7, 14, and 28 days. The flow chart in Figure 2 shows how we introduced the AAM to the LODR prediction using the 1D CNN.

## 4. Results

### Discussion of the Results

To validate our LOD predictions, we included an error analysis of the differences between the predictions and the final results obtained from the observational data. This analysis revealed that the prediction errors using different input sizes increased gradually with the increase in the input size. Furthermore, by comparing the LODR + AAM with the LODR, similar patterns and behaviors became obvious for all the test cases evaluated, specifically noting that the prediction accuracy increased after introducing the AAM. Table 1 shows the MAE of the ultra-short-term prediction using 7 days, 14 days, and 28 days as input before and after introducing the AAM.

**Figure 7:**
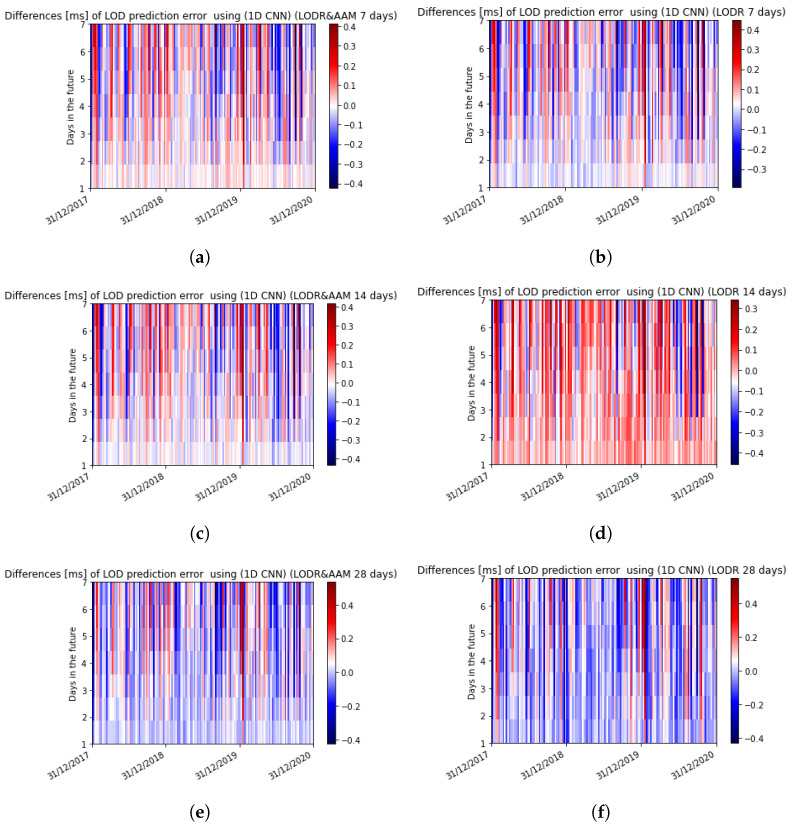
Errors of the predicted LOD with 7, 14, and 28 prior days before and after introducing the AAM using the 1D CNN. (**a**) LOD prediction error using 7 prior days after introducing the AAM; (**b**) LOD prediction error using 7 prior days; (**c**) LOD prediction error using 14 prior days after introducing the AAM; (**d**) LOD prediction error using 14 prior days; (**e**) LOD prediction error using 28 prior days after introducing the AAM; (**f**) LOD prediction error using 28 prior days.

**Figure 8:**
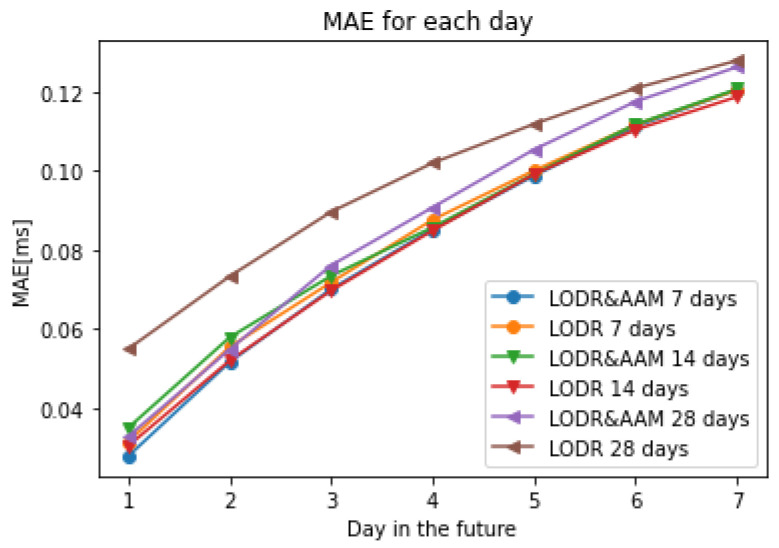
Mean absolute errors of the predicted LOD using the 1D CNN before and after introducing the AAM with different input sizes (7, 14, 28 days).

The presented technique showed an MAE larger than 0.1 ms after the 6-day prediction using 7 and 14 prior days as inputs, whereas the use of 28 prior days reached the aforementioned error one day earlier. A visual inspection of the differences between the observed and predicted data between December 2017 and December 2020 for all the input sizes revealed that the use of 7 prior days produced the best prediction results for the first and second days with MAEs of 0.027 ms/day and 0.051 ms/day, respectively (Figure 7 and Figure 8).

**Figure 9:**
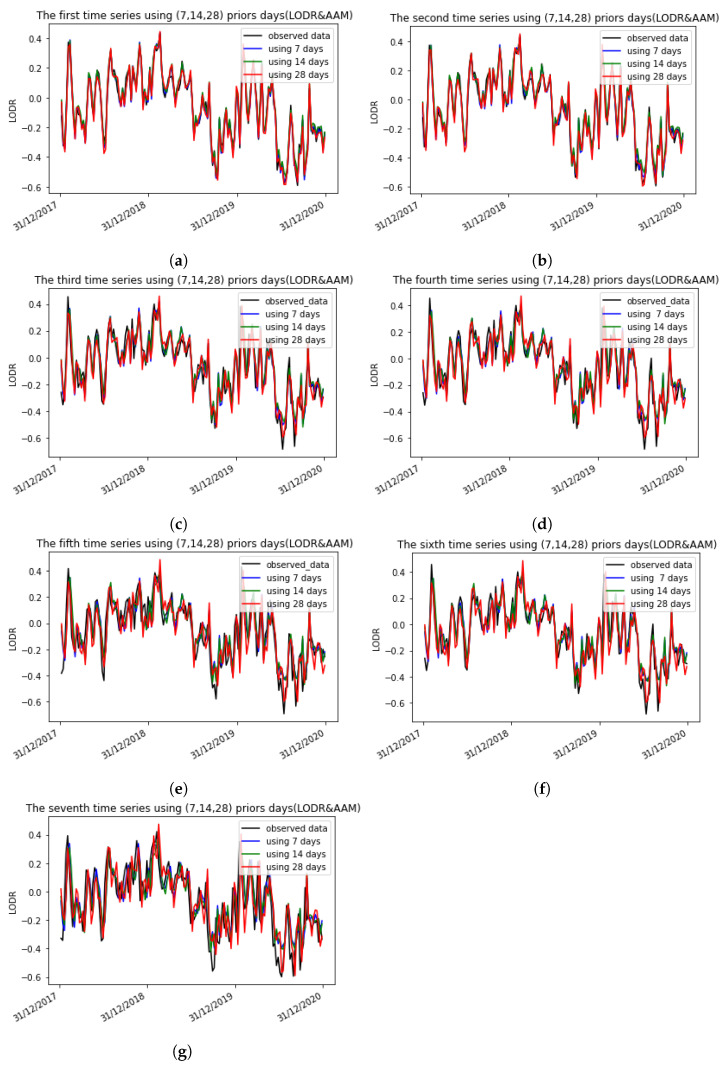
The seven time series using 7, 14, and 28 prior days with the LODR + AAM using the 1D CNN. Each plot represents, respectively, the first and the second to the seventh prediction days using different input sizes before and after introducing the AAM. The number of predicted weeks covers 3 years (2017, 2018, 2019). (**a**) The first time series using 7, 14, 28 prior days (LODR + AAM); (**b**) The second time series using 7, 14, 28 prior days (LODR + AAM); (**c**) The third time series using 7, 14, 28 prior days (LODR + AAM); (**d**) The fourth time series using 7, 14, 28 prior days (LODR + AAM); (**e**) The fifth time series using 7, 14, 28 prior days (LODR + AAM); (**f**) The sixth time series using 7, 14, 28 prior days (LODR + AAM); (**g**) The seventh time series using 7, 14, 28 prior days (LODR + AAM).

**Figure 10:**
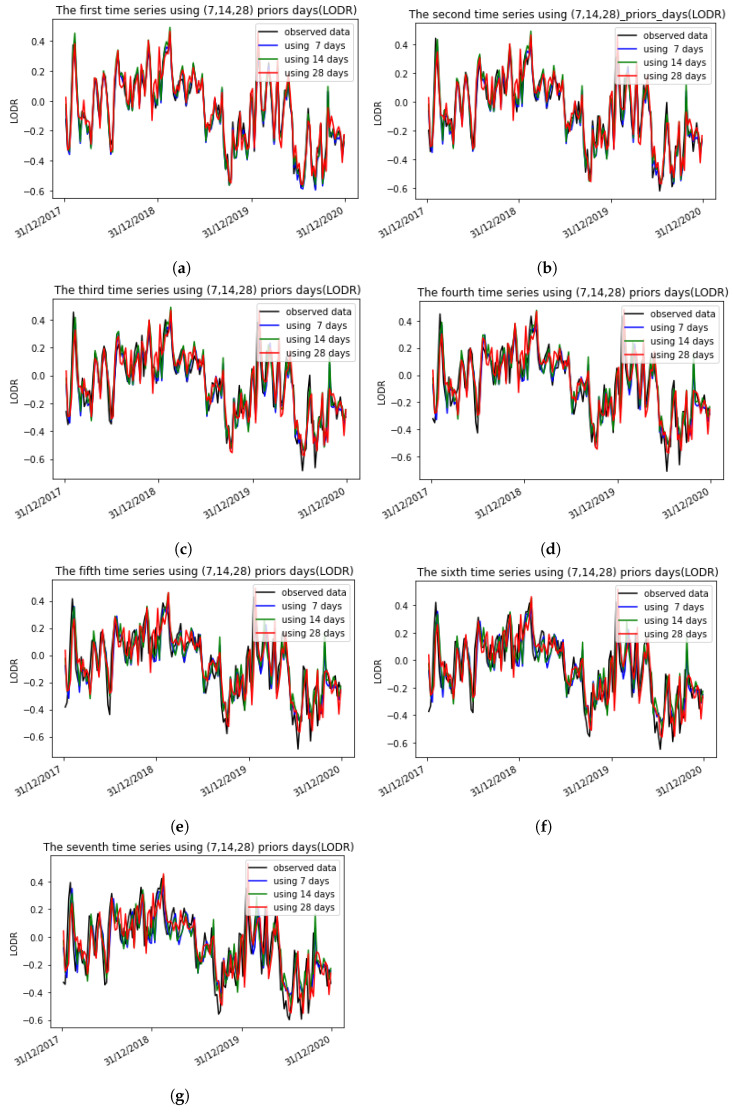
The seven time series using 7, 14, and 28 prior days with the LODR using the 1D CNN. Each plot represents, respectively, the first and the second to the seventh prediction days using different input sizes. The number of predicted weeks covers 3 years (2017, 2018, 2019). (**a**) The first time series using 7, 14, 28 prior days (LODR); (**b**) The second time series using 7, 14, 28 prior days (LODR); (**c**) The third time series using 7, 14, 28 prior days (LODR); (**d**) The fourth time series using 7, 14, 28 prior days (LODR) (**e**) The fifth time series using 7, 14, 28 prior days (LODR); (**f**) The sixth time series using 7, 14, 28 prior days (LODR); (**g**) The seventh time series using 7, 14, 28 prior days (LODR).

In addition, the proposed algorithm was also capable of capturing the periodic terms in the LODR residual time series. This was confirmed by the representations of the seven time series and the observed data with 7, 14, and 28 prior days before and after introducing the AAM using the 1D CNN, as seen in Figure 9 and Figure 10, where each plot represents, respectively, the first and the second to the seventh predicted days using different input sizes before and after introducing the AAM. The number of predicted weeks covers 3 years (2017, 2018, 2019).

**Figure 11:**
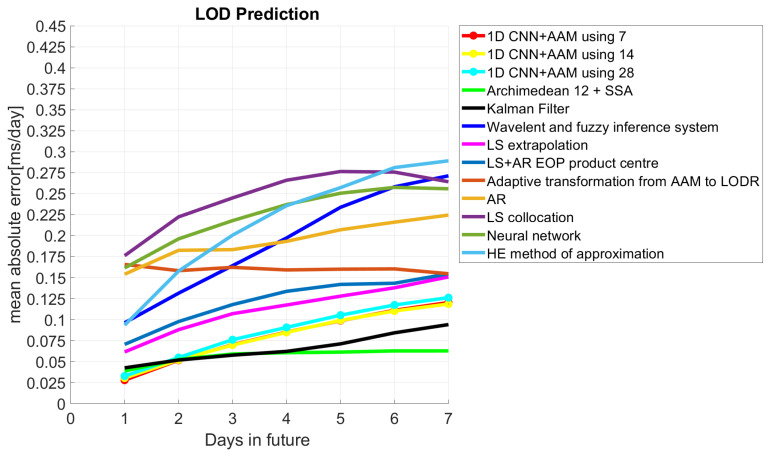
Mean absolute errors of the predicted LOD using the 1D CNN and the 2ns EOP PCC results.

The first Earth orientation parameter prediction comparison campaign, the EOP PCC, gave us the chance to compare our results with the highest number of existing prediction methods used for EOP predictions. The results of the first EOP PCC were kindly provided by Kalarus [37] to one of the authors and are the same as those used in [5]. Although we tried our best to meet the same conditions as those of the first EOP PCC to show the presented method’s effectiveness, the EOP PCC participants used the last values (predictions) with lower precision compared to the a posteriori data (“finals”). In any case, in a situation of real competition such as the current EOP PCC2, any prediction method applied to the LOD should be subject to the same limitations and faces the same problem with using predicted values. Note that the WRMS values of the EOP PCC were obtained when EOP 05 C04, which was discontinued in 2012, was the conventional IERS daily solution. However, this fact has no significant impact on the comparison of the LOD predictions, as shown, e.g., in [39]. Taking into account this limitation, we compared the prediction results with the existing techniques of the first EOP PCC, showing that the proposed approach can efficiently predict the LOD (Table 2). We only predicted 7 days in the future because we introduced the Z-AAM from the GFZ, which is predicted every 7 days. As we can see in Figure 11, it was the most accurate technique for the first prediction day, independent of the use of different input sizes (with an MAE of about 0.30 ms). It is important to note that the use of the AAM and 7 days as the a priori values obtained the best errors for the second-day prediction (0.051 ms), which makes these results better than the Copula Archimedean 12+SSA and Kalman filter. The comparison with the rest of the prediction techniques (i.e., wavelet, LS extrapolation, LS+AR, adaptive transformation, AR, LS, NN, and HE (harmonic and exponential method of approximation)) also showed smaller errors between the first and seventh prediction days. Lastly, the Copula + SSA models in [5] showed comparable performance with our technique, even with a smaller MAE for the first 4 days in the future compared to the Joe + SSA approach, and were better than the Frank + SSA approach for the first 3 days in the future.

## 5. Conclusions

In this study, a combination of stochastic and deterministic methods was studied for LOD predictions. LS was used as a deterministic technique to obtain stochastic residuals (the difference between the observed data and the LS model). Consequently, a one-dimensional convolutional neural network (1D CNN) was used to predict the time-varying behaviors of the LOD change using different input sizes (i.e., 7, 14, and 28 days). Considering that the axial AAM function is strongly correlated with the LOD change, we also introduced it into the length-of-day data with the tide model removed (LODR) to predict the LOD change. The results showed that after introducing the AAM, the prediction accuracy improved in all the tested cases, especially using 28 prior days as the input size, with an improvement of about 41% for the first prediction day. The comparison of the results after introducing the AAM with different input sizes showed that using 7 prior days resulted in a sophisticated performance with low errors on the first and second days (0.027 and 0.051 ms, respectively). The comparison with respect to the first EOP PCC indicated that the 1D CNN can precisely predict the LOD parameters of ultra-short predictions (from 1 to 7 days), providing comparable accuracy, and is even better than the Kalman filter and Copula + SSA methods for the first and second days. Regarding the potential application of the proposed method to provide operational LOD predictions, we had to account for the latency associated with the availability of the VLBI LOD estimates (approximately one month). This drawback could be solved by feeding our method with official and available predictions, for instance, the USNO finals.daily series. This strategy was typically utilized in the first/second EOP PCC.

Based on these results, the LOD forecast can be considerably improved using the 1D CNN technique. In spite of the reality that we set up this technique with a single output, much more needs to be done. We need to make greater efforts to build models using a multi-output strategy to predict more than one parameter. In addition, we should find new variables to include as a priori data in 1D CNN models. These new variables could improve the results for short/mid/long-term predictions. Finally, the values of the effective angular momentum (EAM) functions could be included in 1D CNN models. In this way, each input series can be handled by a separate CNN and the output of each of these submodels can be combined before a prediction is made for the output sequence.

## Figures and Tables

**Table 1:** Comparison of the mean absolute errors of the predicted LOD using the 1D CNN before and after introducing the AAM using different input sizes (7, 14, 28). Units: (ms/day).

	Using 7 Days		Using 14 Days		Using 28 Days	
**Day**	**LODR**	**LODR + AAM**	**LODR**	**LODR + AAM**	**LODR**	**LODR + AMM**
Day 1	0.031	0.027	0.035	0.030	0.055	0.032
Day 2	0.055	0.051	0.057	0.052	0.073	0.054
Day 3	0.071	0.070	0.073	0.069	0.089	0.076
Day 4	0.087	0.085	0.085	0.084	0.101	0.090
Day 5	0.10	0.098	0.0992	0.099	0.111	0.105
Day 6	0.116	0.115	0.111	0.110	0.120	0.117
Day 7	0.1203	0.1204	0.12	0.118	0.127	0.12

**Table 2:** Comparison of 1D CNN prediction and 2ns EOP PCC prediction errors. Units: (ms/day).

Prediction Day	1	2	3	4	5	6	7
1D CNN + AAM using 7 days	0.027	0.051	0.070	0.085	0.098	0.115	0.1204
1D CNN + AAM using 14 days	0.030	0.052	0.069	0.084	0.099	0.110	0.118
1D CNN + AAM using 28 days	0.032	0.054	0.076	0.096	0.105	0.117	0.12
Archi 12 + SSA	0.47	0.060	0.063	0.063	0.063	0.064	0.066
Kalman filter	0.042	0.051	0.057	0.062	0.071	0.084	0.094
wavelet	0.096	0.131	0.164	0.197	0.233	0.258	0.271
LSE	0.061	0.088	0.107	0.117	0.128	0.138	0.151
LSE+AR EOP PC	0.070	0.097	0.118	0.133	0.142	0.143	0.154
Adaptive transform	0.165	0.158	0.162	0.159	0.160	0.160	0.160
AR	0.154	0.182	0.183	0.193	0.207	0.216	0.224
LSC	0.176	0.222	0.245	0.266	0.276	0.275	0.264
NN	0.61	0.196	0.218	0.237	0.250	0.257	0.256
HE	0.093	0.157	0.200	0.235	0.257	0.281	0.289

## Data Availability

Data is available upon request to correspondence author.
